# Enrichments Adjusted to the Number of Litters: Simple Approach to Improve the Welfare of Weaned Piglets under Farm Conditions

**DOI:** 10.3390/ani14202972

**Published:** 2024-10-15

**Authors:** Nejc Kuri, Janko Skok, Dejan Škorjanc, Maja Prevolnik Povše

**Affiliations:** Faculty of Agriculture and Life Sciences, University of Maribor, Pivola 10, 2311 Hoče, Slovenia; nejc.kuri@student.um.si (N.K.); janko.skok@um.si (J.S.); dejan.skorjanc@um.si (D.Š.)

**Keywords:** pig, weaning, welfare, aggression, behaviour, injuries, environmental enrichment

## Abstract

Environmental enrichment is one of the methods used to reduce conflicts at weaning, which erupt when mixing unknown litters and are known to negatively affect growth and welfare of piglets. Here we aimed to test a simple approach of increasing the amount of existing enrichment objects to mitigate post-weaning aggression. The experiment comprised ten groups of piglets (five controls, five tests) with 3–4 litters weaned in each of them. In the control group, a red hanging plastic object (biting ring) and a piece of spruce wood (7 cm × 7 cm × 30 cm) were used for enrichment. In the test group, the enrichment was multiplied according to the number of weaned litters (one toy and one piece of wood was provided per each mixed litter). On days 1, 3, 5, 7 and 14 after weaning, behaviours (negative social behaviours, pen equipment-directed and enrichment-directed behaviours) and injuries (on different parts of the body, tail lesions, lameness) were recorded. On day 21 after weaning, the overall welfare status of the piglets was assessed using the Welfare Quality^®^ protocol for pigs. A significant (4-fold) increase in the enrichment-directed behaviours was observed in the test group (*p* < 0.001). Consequently, there were 3.5 times fewer negative social behaviours (*p* < 0.001), 38–52% fewer injuries on different body parts (*p* < 0.001) and higher overall welfare status of the piglets in the test group. Adjusting enrichment to the number of weaned litters proved to be an effective solution to reduce aggression at weaning and improve piglets’ welfare. The study provides important information on the use of enrichment material in commercial weaner pens and demonstrates the possibility of significantly improving pig welfare with low effort, input and cost.

## Simple Summary

Weaning is one of the most stressful phases in intensive pig production. The mixing of unknown litters initiates the establishment of a new social order through conflicts, which are abnormally increased under commercial farming conditions due to several other sudden changes in the piglets’ environment, such as the separation of the piglets from the sow and changes in feeding and space. Providing appropriate environmental enrichment is a possible solution to mitigate stress and aggression after weaning. In the present study, we used a combination of toys and pieces of wood as enrichment and adjusted the number of enrichment objects to the number of weaned litters. In the control group, one toy and one piece of wood were provided for the entire weaning group, while in the test group, one piece of wood and one toy were provided per each mixed litter. The piglets from more enriched pens showed more enrichment-directed behaviours and consequently less negative social and damaging behaviours. Adjusting enrichment to the number of weaned litters proved to be an effective solution to reduce aggression and improve the welfare of weaned piglets. It is also easily applicable in commercial facilities, cost-effective and of sustainable interest.

## 1. Introduction

Environmental enrichment is a broad term used to describe changes, modifications or additions to the environment of animals that improve their living conditions by encouraging the expression of a broader range of normal behaviours [1]. In pigs, species-specific behaviour includes rooting, chewing, biting, etc. [2,3]. In a barren environment with little or no opportunity for exploratory behaviour, pigs focus their curiosity on other pigs and equipment, leading to stereotypies and harmful behaviours [4], especially in crowded commercial pens. The use of environmental enrichment helps to create a more complex and interactive environment that provides pigs with challenges and novelties that simulate situations that occur in the wild or that allow for the expression of species-specific behaviours [5].

Providing enrichment to pigs could also contribute to mitigation of aggressive behaviours that could arise at various stages of rearing [6,7,8]. Exaggerated aggression occurs at/after weaning under commercial rearing conditions. This is known to be one of the most critical phases in the life of pigs, as it involves the separation of piglets from the sow, mixing of unknown litters, a change in feeding, and an altered social and physical environment. These changes occur slowly and spontaneously in the natural environment, but are suddenly introduced in intensive farming, leading to an outbreak of aggression with undesirable consequences for the pigs and for the production [9,10]. Different methods have been tested to minimise stress and conflicts around weaning (e.g., early socialisation [11]), with environmental enrichment being one of them [12,13,14,15,16].

In the EU, access to environmental enrichment is prescribed in Council Directive 2008/120/EC [17]. According to this, group-housed pigs should have constant access to bedding or other materials that allow exploration and occupation. Comprehensive research over the past decades has led to the insights that not all materials/objects provided to pigs are equally effective in improving animal welfare (e.g., [1,2,18,19,20]. Based on that, the EU Commission has issued recommendations [21] on the adequacy of enrichment material. To be effective, enrichment material should be edible, chewable, investigable, and manipulable/deformable. It should also be of sustainable interest, accessible, available in sufficient quantity, clean and safe [21]. The ideal material for pigs is straw because it satisfies all previously mentioned recommendations. Straw is an important stimulus and outlet for exploration, rooting and chewing and also provides physical and thermal comfort, but is incompatible with prevailing slatted systems and/or not always available [22]. Therefore, in pigs, point-source enrichment objects such as ropes, chains, wood, tyres, plastic objects, etc., play an important role in this respect.

Although legally required in the EU, enrichment is not consistently used in practice. In addition, ineffective solutions are often observed on farms that do not meet the needs of the pigs, but appear to be provided only to meet certain mandatory or voluntary requirements [23,24]. Recent critical reviews have also shown that the transfer of scientific knowledge into practice has so far not been sufficient and urge provision of new materials and forms of enrichment that are optimal for practice [23,25].

As it is well known that littermates form a more socially cohesive subgroup even after weaning [26,27], we tried to improve the welfare of weaned piglets by adjusting the existing enrichment (used on the farm under study and also widely used elsewhere in commercial pig farming) to the number of weaned/mixed litters by providing one set of objects for each mixed litter. We hypothesised that weaners in the group with adjusted enrichment would exhibit less negative social behaviours and damaging behaviours and generally obtain better welfare scores than weaners from the control group in which only one set of enrichment objects was provided for the entire weaning group.

## 2. Material and Methods

### 2.1. Experimental Unit, Pens and Animals

The experiment was conducted on a commercial pig farm in north-eastern Slovenia from February to May 2023 in accordance with the applicable animal welfare regulations [17]. It was conducted under routine farm conditions; the changes made during experiment did not require ethics committee approval. The study comprised 35 litters with 363 piglets (Swedish Landrace × Large White). During lactation, all piglets had the same rearing conditions and handling. Solid feed was available to the piglets from the 7th day after birth. Piglets had free access to water. Surgical castration, tail docking and teeth clipping were performed within the first day after birth without anaesthesia or analgesia. Cross-fostering was not performed in any of the litters.

**Figure 1:**
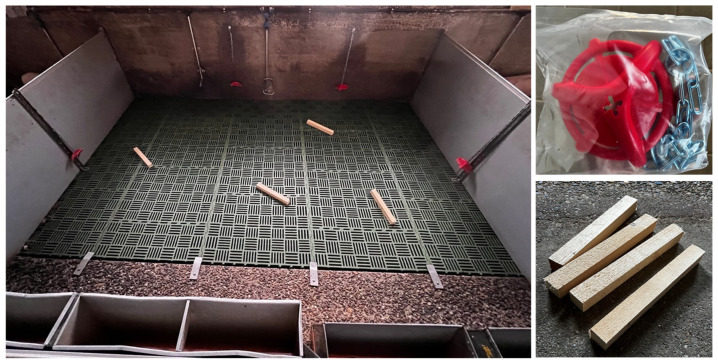
Enrichment objects used in the experiment (toy, wood) and an example of the test pen.

Weaning was performed at the age of 28–35 days. In our experiment, three or four litters were weaned together and placed in one weaning pen. The number of mixed litters depended on the farm conditions at the time, i.e., the number of litters available for weaning in a given period. Five weaning pens were available in the experimental facility, four of which had a size of 9.1 m^2^ (3.5 m × 2.6 m) and one of 6.2 m^2^ (2.4 m × 2.6 m). Although one pen was smaller, the space availability was in accordance with the legal requirements [17]. None of the pens had access to an outdoor area (Figure 1). The pens were separated from each other by 100 cm high, solid plastic side walls. The average room temperature was 26 °C and was regulated by a ventilation system. Each pen was equipped with a heating plate. The pens had a metal slatted floor covered with perforated plastic material. A combination of daylight and artificial light was used to ensure 14 h of light (at least 40 lx) per day. The piglets were fed ad libitum; feeders were filled each day around 8 a.m. with a home-prepared balanced creep-feed mixture for weaned piglets, consisting of home-grown maize and barley as well as purchased soy and mineral-vitamin supplement. The transition from the starter feed to the feed for weaned piglets was carried out gradually over a period of one week. Piglets in each pen had free access to water through two nipple drinkers and one drinking cup. Each pen was equipped with four feeding troughs—two large and two small rectangular grey sheet-metal. The large troughs were 95 cm long, 25 cm wide and 15 cm deep, while the small were 61 cm long, 20 cm wide and 12 cm deep.

### 2.2. Experimental Design

A total of ten groups of weaned piglets were included in the experiment and were divided into the five control and five test groups. Each of the five available weaning pens was used twice during the experiment (two batches)—once as a control and once as a test pen (Table 1). Pens 2, 4 and 5 were used as controls in the first batch and for the test group in the second batch. Conversely, pens 1 and 3 were used in the first batch for the test and in the second batch for the control group. In the control group, one piece of wood and one toy were used, i.e., enrichment that is used on the farm in daily breeding practice (Figure 1). In the test group, the number of enrichment objects was multiplied according to the number of weaned litters, i.e., by providing one set of objects for each mixed litter (e.g., when four litters were mixed, four pieces of wood and four red toys were added in the test pen). The number of piglets in the weaning groups varied between 30 and 47 (on average 36 ± 6; Table 1). In total, there were 187 and 176 piglets in the control and test groups, respectively. The toys used were commercially available red plastic objects (pig biting rings with a diameter of 15 cm) hanging on 60 cm long chains attached to the wall 80 cm above the floor and evenly distributed in the pen (Figure 1). In addition, freshly cut pieces of spruce wood (7 cm × 7 cm × 30 cm) were added on the floor of all pens. Enrichment objects were installed before the beginning of the experiment and were not replaced during experimental period (21 days). The number of piglets per enrichment object was ~5 in the test and ~20 in the control group (Table 1).

### 2.3. Measurements and Observations

The measurements were carried out according to the Welfare Quality^®^ protocol for pigs [28] and were divided into three sets: (i) piglet behaviours—negative social behaviours (agonisms), enrichment- and pen equipment-directed behaviours; (ii) injuries—on different parts of the body, tail lesions and lameness; and (iii) overall welfare assessment/status, which comprised assessment of feeding, housing, health and behaviour of pigs. Behaviours and injuries were assessed six times during the observation period, on days 1, 3, 5, 7, 14 and 21 after weaning. All observations were made concerning individual animals and then considered at pen level (expressed as the number per pig). The overall welfare status was assessed at the end of the experiment (day 21 after weaning).

(i) Piglet behaviours

The observations were carried out in the morning one hour after feeding, when the animals were most active. The observer entered the room, made sure that all the animals were up and waited a few minutes for the piglets to acclimate to him. The behaviours were assessed from the passageway in five consecutive scans of approximately half a minute per scan with an interval of 2 min between scans. All five scans were combined for further analysis. The following types of behaviours were recorded:

- enrichment-directed: exploring the enrichment material defined as play or investigating towards the enrichment objects (with nose/head directed towards the enrichment material—sniffing, nosing, chewing, biting or any other manipulation such as pushing, moving around etc.);
- pen equipment-directed: exploring pen defined as sniffing, nosing, licking or chewing equipment of the pen;
- negative social: aggressive interactions between piglets, including fighting, biting, pushing or any other social behaviours with a response from the disturbed animal (fighting back or running away).

(ii) Injuries

The injuries (on different body parts, tail lesions and lameness) were recorded immediately after the behavioural observations.

According to the protocol [28], injuries are defined as surface penetration of the epidermis (e.g., scratches) or as penetration of muscle tissue (e.g., wounds). To assess injuries, the assessor entered the pen and assessed each piglet from a distance of 0.5 m. Injuries were assessed separately on five body regions: ears, front (head to back of shoulder), middle (back of shoulder to hind-quarter), hind-quarter and legs. The tail area was not considered here. For further analysis, we specified only three body parts: ears, front part and middle-back part. Due to their low incidence, injuries on the middle region and hind quarter were combined into one part, i.e., the middle-back part. Injuries on the legs were extremely rare and were not statistically analysed. We distinguished between two categories of injuries: mild (scratches longer than 2 cm or two parallel scratches up to 0.5 cm apart or small wounds of less than 2 cm) and severe (bleeding wounds of more than 2 cm or healed wounds of more than 5 cm). On each observation day, the assessor counted all injuries on the piglets’ skin without distinguishing between fresh and older injuries.

The damage to the tail caused by bites can range from superficial bites on the tail to the absence of the tail. To assess tail biting, it was important that all animals stood upright so that the observer had a clear and unobstructed view of the pigs’ tails. The severity of tail biting was assessed using the Welfare Quality^®^ [28] scale: score 0 if there were no signs of tail biting or superficial biting without fresh blood and swelling (reddish areas on the tail were not considered wounds unless fresh blood was present), and score 2 if there were obvious signs of tail biting—blood, swelling, infection, missing parts of the tail tissue or scabs. In this measurement, the number of piglets with clear signs of tail biting was determined (score 2).

Lameness, i.e., the inability to use one or more legs in a normal manner, can range from limited mobility or inability to bear weight to total recumbency. To assess lameness, the assessor ensured that the piglets walked a certain distance. A clear and unobstructed view of the moving animal was also crucial. The assessor evaluated pig’s walking using the Welfare Quality^®^ scale [28]: score 0 (no lameness) for normal walking or difficulty walking but still using all four limbs, or swaying of caudal body while walking or shortened stride, score 1 (mild lameness) for minimal weight bearing on the affected limb and score 2 (severe lameness) for no weight bearing on the affected limb or inability to walk.

(iii) Overall welfare assessment of piglets

The overall assessment of welfare was carried out on the 21st day after weaning (Table 2). The appropriateness of feeding, housing, health and behaviour was assessed using 12 independent criteria (2–4 per principle), which were evaluated with ~30 measurements and observations directly in the barn/pen. In our study, the last criterion on emotional states was not assessed. The details of overall welfare assessment can be found in the Welfare Quality^®^ protocol [28].

### 2.4. Statistical Analysis

The statistical analyses were carried out using SPSS software, version 29. For repeated measurements (behaviours, injuries), the effect of treatment and its temporal dynamics on these variables was first analysed. The response variables were analysed using a linear mixed model with the predictor variables group (control, test) and time (day 1, 3, 5, 7, 14, 21) and their interaction. Pen (1–5) was added in the model as random effect and space per pig as covariate to account for a different number of pigs per pen and different pen size. Weaning group (1–10) was specified as subject to account for repeated observations of the same group/piglets on different days (repeated = time). Estimated means with standard errors of mean for the factors and interactions analysed were calculated for the generated models. Post hoc comparisons were adjusted using the Bonferonni correction for multiple testing. The model described above was applied to enrichment- and pen equipment-directed behaviours, injuries on ears, front and middle/back part of the body. These traits were normally distributed (tested with the Shapiro–Wilk test) and/or visual inspection of the residual plots for the models revealed no obvious deviations from homoscedasticity or normality. For negative social behaviours and tail lesions, the nonparametric Mann–Whitney U test was applied to test for differences between control and test group overall and by observation day as these two traits were not normally distributed (many zeros in the data, especially in the test group). In the case of lameness and injuries on legs (which were also repeatedly assessed during observation period), only descriptive statistics were reported due to the very low incidence of these injuries.

For five indicators of the overall welfare assessment (absence of hunger, absence of injuries, social behaviours, other behaviours, human–animal relationship), the median and quartiles were calculated for the control and test groups and the differences between the control and test group were tested using the non-parametric Mann–Whitney U test as the variables were not normally distributed. For the remaining six indicators of the overall welfare assessment (absence of thirst, comfort at resting, thermal comfort, ease of movement, absence of diseases, absence of pain), the results are presented descriptively and were not statistically analysed as there were no variation between the groups.

## 3. Results

### 3.1. Behaviours

**Figure 2:**
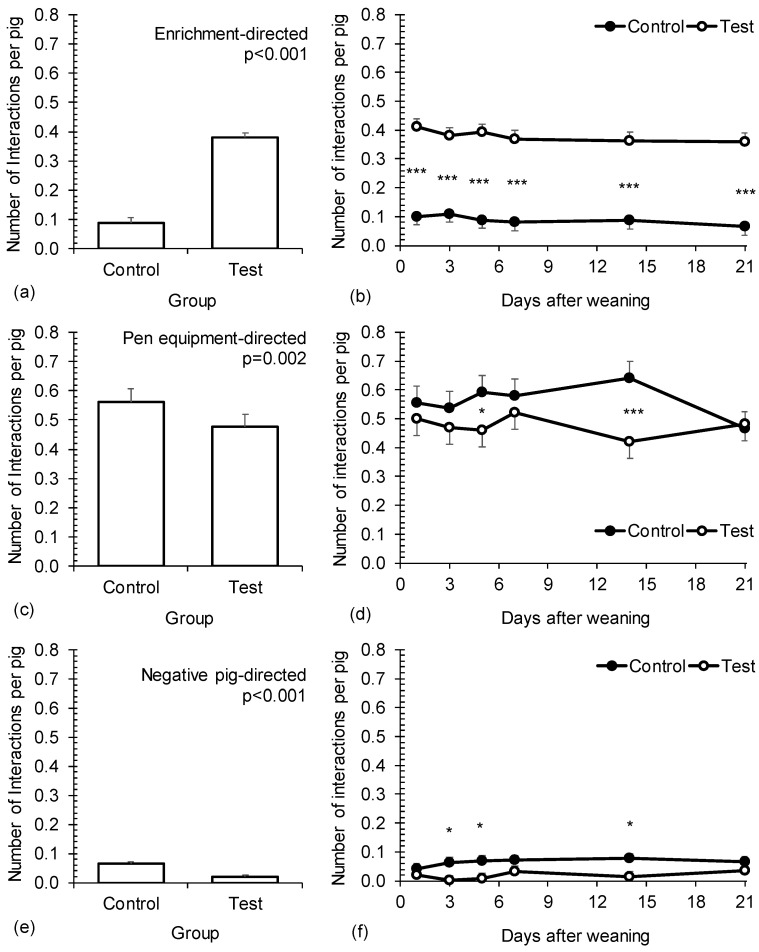
The effect of treatment and time on the enrichment-directed behaviours (**a**,**b**), pen equipment-directed behaviours (**c**,**d**) and negative social behaviours in piglets (**e**,**f**). Control—one set of enrichment objects (one piece of wood and one toy) provided for the whole weaning group of piglets. Test—quantity of enrichment objects adjusted to the number of litters mixed (one piece of wood and one toy provided per each litter). The effect of time was not significant in any model (*p* ≥ 0.05). The effect of interaction treatment group × time was not significant in any model (*p* ≥ 0.05). *, *** denote statistically significant differences between groups within day (*p* < 0.05 and *p* < 0.001, respectively).

The effect of treatment on piglets’ behaviours and its temporal dynamics is presented in Figure 2. Behaviours were significantly affected by treatment but not by time or their interaction. Enrichment-directed behaviours were four times more frequent in the test than in the control group (0.38 and 0.09 per pig, respectively; *p* < 0.001; Figure 2a). The difference between the control and test group was constant over the whole observation period and statistically significant on all observation days (*p* < 0.001; Figure 2b). In case of pen-directed behaviours, the difference between the groups was smaller (~15%), but significant (*p* = 0.002; Figure 2c). Less occupation with the pen equipment was observed in the test compared to the control group (0.48 and 0.56, respectively). The same pattern was observed on each observation day (i.e., 10–35% lower frequency in the test group) except on day 21, where there were no differences between the groups (Figure 2d). Negative social behaviours were in general rare, but were significantly less frequent in the test compared to the control group overall (0.02 and 0.06 per pig, *p* < 0.001; respectively; Figure 2e) and also on certain observation days (*p* < 0.05; Figure 2f).

### 3.2. Injuries

**Figure 3:**
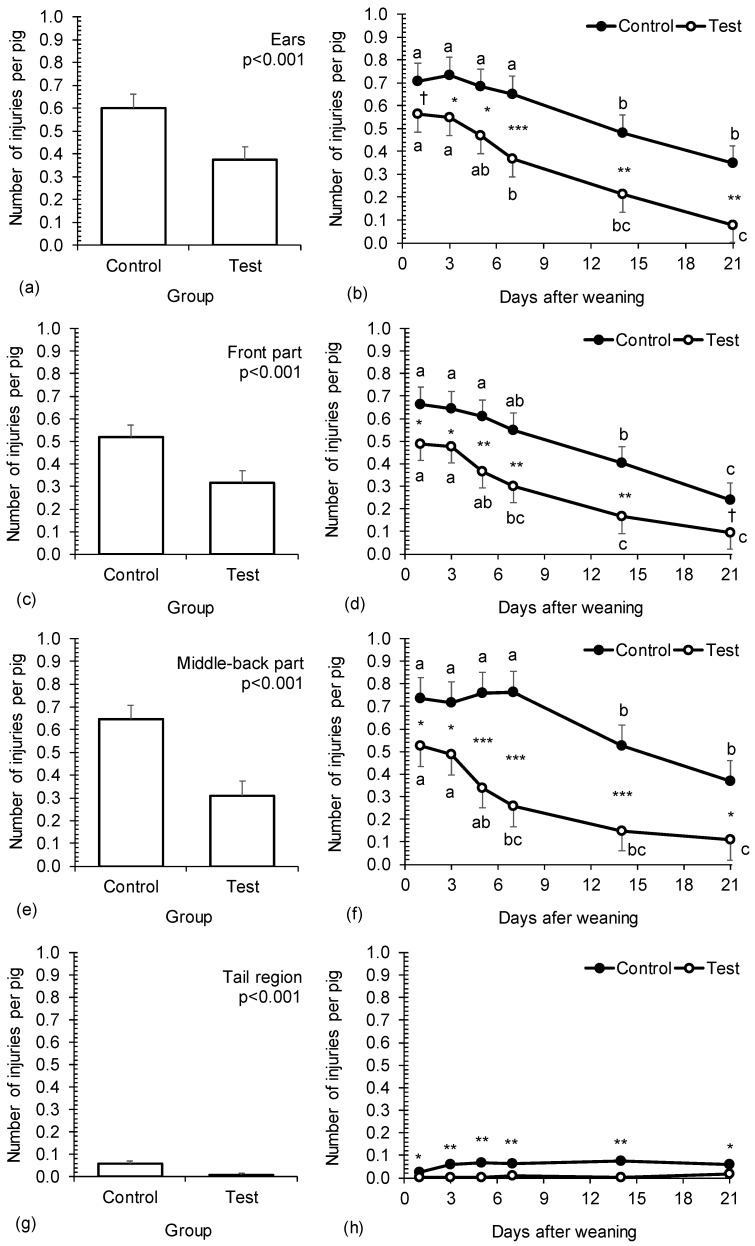
The effect of treatment and time on the incidence of injuries on ears (**a**,**b**), front part (**c**,**d**), middle-back part (**e**,**f**) and tail region (**g**,**h**) in piglets. Control—one set of enrichment objects (one piece of wood and one toy) provided for the whole weaning group of piglets. Test—quantity of enrichment objects adjusted to the number of litters mixed (one piece of wood and one toy provided per each litter). The effect of time was highly significant (*p* < 0.001) in all models. The effect of interaction treatment group × time was not significant in any model (*p* ≥ 0.05). *, **, *** denote significant differences between groups within day (*p* < 0.05, *p* < 0.01 and *p* < 0.001, respectively); ^†^ denotes the tendency of significant differences between groups within day (0.05 ≤ *p* < 0.10); ^abc^ denote statistically significant differences between days within treatment group.

Injuries on different body parts. All injuries recorded in our experiment were classified as mild (no severe cases were observed). Injuries are presented for three body parts: ears, front and middle-back. Injuries on legs were almost absent (21 injuries in total, all in the control group). The effect of treatment on injury prevalence and its temporal dynamics are shown in Figure 3. In general, injuries were strongly influenced by treatment and time, but not by their interaction. Overall, significantly fewer injuries were observed in the test group compared to the control group (*p* < 0.001). The test piglets had 37.9% fewer injuries on the ears, 39.1% fewer injuries on the front part and 51.9% fewer injuries on the middle-back part (Figure 3a,c,e). The differences between the control and test group were evident during the whole observation period and were statistically significant on almost all observation days. In both the control and test groups, injuries in all body parts decreased significantly during the experimental period; by 49.8–63.8% in the control group and by 79.4–85.9% in the test group, resulting in very low levels in the test group at the end of experiment. In the test group, a significant reduction in injuries was already observed in the first week, which was not the case with the control group (Figure 3b,d,f).

Tail lesions. Significant effect of treatment was observed on tail lesions overall and by observation day (Figure 3g,h). Tail lesions were rare compared to injuries on ears, front, middle and back part of the body, but the results still showed considerably fewer tail lesions in the test group compared to the control (0.01 and 0.06 per pig, *p* < 0.001; respectively). The differences between the control and test group were constant and statistically significant throughout the experimental period (*p* < 0.05).

Lameness. Only a few cases of lameness were recorded in our study. In the test group, only one piglet was assessed as slightly lame during the entire experiment. In the control group, there were five cases of severe lameness. All were observed in the first week after weaning and then returned to normal by the end of the study.

### 3.3. Overall Welfare Assessment

The overall assessment of piglets’ welfare at the end of experiment is shown in Table 3. According to the results, the feeding and housing principles were equivalent in both groups. There was no difference between groups in terms of percentage of lean piglets (median of 2.7 and 0.0, respectively). The indicators for water supply were the same in both groups. Each pen had three drinkers with one drinker available per 10–15 piglets. All drinkers were kept clean during the experiment. No signs of discomfort at resting or of thermal discomfort were observed in either group. The floor area available for the piglets was also the same in both groups (~1.5 m^2^/100 kg body weight). In the health principle, significant differences between the control and test group were only observed for the criterion related to injuries. Significantly more injuries were observed on different body parts including tail lesions in the control than in the test group. Lameness was not observed in any of the pens at the end of the experiment. Regarding the absence of disease, the warning threshold was exceeded for mortality only (threshold of 2.6%). It was exceeded in two weaning groups in the control (with 3% and 4% mortality) and in one weaning group in the test group (with 3% mortality). In total, 3 out of 187 piglets in the control group and 1 out of 176 piglets in the test group died during experiment. No other health problems/symptoms were detected. Castration and tail docking are regularly carried out on the farm without anaesthesia. In the principle of behaviour, significant differences between groups were found, but only in the enrichment-directed behaviours which was more frequent in the test group. Control and test groups did not differ in the pen equipment-directed and negative social behaviours and in the percentage of piglets showing a panic reaction.

## 4. Discussion

Increasing the amount of enrichment by providing one set of enrichment objects to each weaned litter significantly increased piglets’ engagement with these objects. Consequently, the piglets paid less attention to their littermates and the pen equipment, resulting in lower levels of negative social and damaging behaviours and improved overall welfare of weaned piglets in a group with additional enrichment. Our hypothesis that post-weaning enrichment should be adjusted to the number of mixed litters rather than to the number of mixed piglets was based on the findings of previous studies [26,27] showing that piglets up to 13 weeks of age are more strongly associated with littermates than with any other category of herd members, i.e., non-littermates, other young animals or adults including their mother. As such it can be considered as an independent entity; thus, when applying such an approach, the litter as a whole is of importance, regardless of its size and structure (sex ratio). Therefore, it should be emphasised that the experimental design applied in our study could not be fully balanced in terms of weaning group size, sex structure, number of piglets per enrichment object, etc.

The overall assessment of animal welfare was mainly carried out to evaluate the general quality of the farming conditions. During the experiment, all conditions and rearing procedures were in line with routine daily practices on the farm; i.e., no changes were introduced except for providing additional enrichment. The differences due to adjusted amount of enrichment were only found in terms of behaviours and injuries. For all other welfare criteria, control and test groups did not differ at all. The minimum standards and requirements were met or even exceeded the threshold for most welfare criteria according to the Welfare Quality^®^ protocol [28]. Therefore, the overall welfare status of the piglets in both groups can be considered satisfactory, although a significant shift towards a higher welfare level was observed in the test piglets due to more enriched pens.

As hypothesised, the addition of as many enrichment objects as there were mixed litters led to a substantial increase in enrichment-directed behaviours and consequently to a decrease in negative social behaviours and pen equipment-directed behaviours during the whole experimental period. We did not distinguish between the toy- and wood-directed behaviours, as the aim of the study was not to test the types/materials of the enrichment objects. According to the assessor, toys were more frequently used by piglets (rough estimate ~70%). In the control group, pen equipment-directed behaviours prevailed (~80%) with enrichment- and negative behaviours being less frequent and of similar frequency. In the test group, extra enrichments changed the proportion of different behaviours, but not their total number. Enrichment-directed behaviours increased to around half, the rest being pen equipment-directed behaviours with negative social behaviours being almost negligible. This is in accordance with findings from the literature stating that when the environment is sufficiently and adequately enriched, pigs direct their attention and curiosity towards enrichment [4,29,30] and can also redirect/modify aggressiveness [6,31]. Otherwise, they tend to focus on other objects/subjects in the vicinity; i.e., pen equipment or body parts of pen mates, the latter being the worst outcome. Furthermore, deficient or insufficient enrichment can represent an additional source of competition for the access to limited resource (i.e., objects) which is a well-known cause of conflicts [32,33] resulting in damaging behaviours, which was very likely the case also in our control group. It should further be noted that the frequency of manipulations with enrichment materials alone is not necessarily a good indicator/predictor of less damaging behaviours—Telkänranta et al. [34], for example, reported similar manipulation with wood and plastic pipes but only wood reduced biting behaviour. However, in our study, a higher frequency of enrichment-directed behaviours resulted in lower levels of damaging behaviours. It is known that post-weaning aggression is usually directed at body parts other than the tail [31,35]. Accordingly, we observed most injuries on the ears, the front and the middle/back part of the body. Yet, on these body parts, significantly fewer injuries were observed in the test group. All these injuries decreased over time in both groups, which was due to gradual establishment of a new social order and thus decreased aggression [36,37,38]. The decrease was stronger in the test group and was evident already in the first week after weaning, when fights for hierarchy are normally most intense [38]. In contrast, tail lesions were rare and constant during the experimental period, although they were less frequent in the test group. The tail lesions were usually a result of non-aggressive biting, which is largely unrelated to hierarchy formation and competition for resources and occurs mainly, though not exclusively, due to the barren environment and unsatisfied need to explore [35]. The low overall level of tail lesions in the present study could also be explained by the fact that the pigs were docked. It is known that tail docking considerably reduces the risk of tail biting in pigs [39].

Our results are consistent with several published studies showing the positive effect of enrichment on animal welfare, although not all studies show this as no effect on welfare is often observed [24]. If this is the case, the object/material cannot be labelled as enrichment, as by scientific definition it should shift the welfare to a higher level. According to the EU Recommendations [21], such enrichment is of marginal interest and should be used in combination with more appropriate enrichments. Additionally, according to the revised four-level outcome/evidence-based classification recently proposed by Taylor et al. [24], such an enrichment is a “pseudo-enrichment”, i.e., an enrichment that may has good intentions but has no positive implications for animal welfare. Accordingly, the enrichment tested in the present study could be classified as “enrichment for meeting basic needs”, as it contributed to a reduction in suffering (fewer injuries on different body parts), but also as “enrichment for pleasure”, as a sustained interest of the piglets is evidenced.

Maintaining interest over a longer period of time is an important prerequisite for environmental enrichment. In our study, we did not observe any decrease in interactions with toys and wood during the 21 days. A similar finding was reported by Telkänranta et al. [34] for fresh wood, where exploratory behaviours in fattening pigs persisted even after 2.5 months of exposure. However, the studies more often demonstrate the lack of sustainable interest; e.g., very quick habituation to all kinds of environmental enrichment was reported by Guy et al. [40], who tested four different objects in a combination of two in growing pigs. The reason for the sustainable interest in our case could be the simultaneous presence of two seemingly different objects, whether different in colour and shape, position, both in terms of height (one at eye level and another at or below the snout) and spatial fixity (one hanging in the same place and another free to move in space), and material (wood and plastic). Although it is often reported that plastic objects are of no or of marginal interest and thus do not contribute to improving welfare [8,23], we still found that they are effective in this regard at least in case of weaned piglets.

In the present study, we proved the effectiveness of enrichment used in adjusted quantity to improve animal welfare. However, to be truly effective, the enrichment must be attractive not only to the animals, but also for the farmers. If they also benefit from it, enrichment has a higher potential to actually be used on farms [19,24]. In our opinion, the tested enrichment material is cost-effective (reasonable price, multiple use, easy cleaning and installation, etc.) and of sustainable interest.

## 5. Conclusions

Enrichment material aligned with the number of mixed litters proved to be an effective solution to mitigate negative consequences of post-weaning aggression. Providing at least as many sets of enrichment objects as the number of litters mixed in a weaning group resulted in a lower potential for damaging behaviours and thus better post-weaning welfare of the piglets compared to the control group receiving one set of enrichment objects. Our findings reveal the need to optimise the quantity of enrichments in a specific situation, and demonstrate that the tested combination of enrichment objects in adjusted quantity is easily applicable in commercial facilities, reasonably priced and of interest to weaned pigs.

## Figures and Tables

**Table 1:** Basic data for individual weaning groups.

Weaning Group	Batch	Pen	Group	No. of Weaned Litters	No. of Piglets (Mortality ^1^)	Space per Pig, m^2^	No. Pigs per Object
1	1	1	Test	3	30	0.30	5.0
2	1	2	Control	4	47	0.20	23.5
3	1	3	Test	3	32	0.28	5.3
4	1	4	Control	3	31	0.20	15.5
5	1	5	Control	4	40 (2)	0.23	19.5
6	2	1	Control	4	38 (1)	0.24	18.8
7	2	2	Test	4	43	0.21	5.4
8	2	3	Control	3	31	0.29	15.5
9	2	4	Test	3	31 (1)	0.20	5.1
10	2	5	Test	4	40	0.23	5.0

^1^ Number of piglets that died during the study.

**Table 2:** Measurements and observations made to assess overall welfare status of piglets at the end of experiment (based on Welfare Quality^®^ protocol [28]).

Principle	Criterion	Measurement/Observation
1 Feeding	1 Absence of hunger	Body conditions—lean piglets
	2 Absence of thirst	Water supply—number of water point, cleanness and functioning of drinkers
2 Housing	3 Comfort at resting	Bursitis, manure on the body
	4 Thermal comfort	Shivering, panting, huddling
	5 Ease of movement	Space allowance, m^2^/100 kg body weight
3 Health	6 Absence of injuries	Lameness, tail biting, wounds on the body
	7 Absence of diseases	Coughing, sneezing, pumping, twisted snout, rectal prolapse, scouring, skin condition, ruptures, hernias, mortality
	8 Absence of pain	Castration, tail docking
4 Behaviour ^1^	9 Expression of social behaviours	Social behaviours (negative interactions)
	10 Expression of other behaviours	Exploratory behaviours (pen-directed interactions, enrichment-directed interactions)
	11 Animal-human relationship	Fear of human—panic reaction

^1^ The last criterion of Welfare Quality protocol^®^—positive motional state—was not assessed within our study.

**Table 3:** Overall welfare status of piglets in the control and test group 21 day after weaning.

Principle	Criterion	Measurement/Observation	Control Group	Test Group	*p*
Feeding	Absence of hunger	% of lean piglets	Me [Q1–Q3] = 2.7 [0.0–7.4]	Me [Q1–Q3] = 0.0 [0.0–3.3]	n.s.
	Absence of thirst	No. of drinking places per pen, no. of piglets per drinker, cleanness of drinkers	3 drinkers/pen, 10–15 piglets/drinker, access to two drinkers, all drinkers clean during experiment	3 drinkers/pen, 10–14 piglets/drinker, access to two drinkers, all drinkers clean during experiment	/
Housing	Comfort at resting	Bursitis	Not observed	Not observed	/
		Manure on the body %	Not observed	Not observed	
	Thermal comfort	Shivering, panting, huddling	Not observed	Not observed	/
	Ease of movement	Space allowance	0.23 m^2^/piglet = 1.5 m^2^/100 kg	0.24 m^2^/piglet = 1.6 m^2^/100 kg	/
Health	Absence of injuries	Lameness	Not observed	Not observed	/
		Injuries—ears	Me [Q1–Q3] = 0.32 [0.28–0.39]	Me [Q1–Q3] = 0.10 [0.05–0.14]	**
		Injuries—front part	Me [Q1–Q3] = 0.24 [0.21–0.29]	Me [Q1–Q3] = 0.09 [0.06–0.11]	**
		Injuries—middle/back part	Me [Q1–Q3] = 0.38 [0.29–0.46]	Me [Q1–Q3] = 0.12 [0.03–0.16]	**
		Tail lesions	Me [Q1–Q3] = 0.06 [0.05–0.08]	Me [Q1–Q3] = 0.00 [0.00–0.03]	*
	Absence of diseases	No. of warning and alarm thresholds exceeded	Warning threshold exceeded once (for mortality)	Warning threshold exceeded twice (for mortality)	/
	Absence of pain	Castration	Yes, all piglets	Yes, all piglets	/
		Tail docking	Yes, all piglets	Yes, all piglets	/
		Anaesthesia	Not applied	Not applied	/
Behaviour	Social behaviours	Negative	Me [Q1–Q3] = 0.06 [0.05–0.09]	Me [Q1–Q3] = 0.00 [0.00–0.08]	n.s.
	Other behaviours	Pen-directed	Me [Q1–Q3] = 0.42 [0.37–0.60]	Me [Q1–Q3] = 0.44 [0.42–0.56]	n.s.
		Enrichment-directed	Me [Q1–Q3] = 0.06 [0.05–0.09]	Me [Q1–Q3] = 0.35 [0.30–0.42]	**
	Human-animal relationship	% of piglets showing panic reaction	Me [Q1–Q3] = 15.6 [3.2–61.0]	Me [Q1–Q3] = 66.6 [25.8–86.7]	n.s.

Control—one set of enrichment objects (one piece of wood and one toy) provided for the whole weaning group of piglets. Test—quantity of enrichment objects adjusted to the number of litters mixed (one piece of wood and one toy provided per each litter). Me—median, Q1—first quartile, Q3—third quartile; n.s.—not significant (*p* ≥ 0.05), *, ** denote significant difference between the control and test group (*p* < 0.05 and *p* < 0.01, respectively).

## Data Availability

The dataset available on request from the authors.
